# A Novel Overall Survival Nomogram Prediction of Secondary Primary Malignancies after Hypopharyngeal Cancer: A Population-Based Study

**DOI:** 10.1155/2022/4681794

**Published:** 2022-04-28

**Authors:** Meng Wan, Dan Zhao, Yan Sun, Weihu Wang

**Affiliations:** Key Laboratory of Carcinogenesis and Translational Research (Ministry of Education/Beijing), Department of Radiation Oncology, Peking University Cancer Hospital and Institute, Beijing 100142, China

## Abstract

**Objectives:**

We aimed to construct a nomogram for predicting the overall survival (OS) of patients with secondary primary malignancies (SPMs) after hypopharyngeal cancer (HPC).

**Methods:**

613 HPC patients were included in the Surveillance, Epidemiology, and End Results (SEER) database between 2000 and 2018, which were divided into training and validation cohorts. The least absolute shrinkage and selection operation (LASSO) and stepwise Cox regression were used to determine the variables by which a nomogram model was established.

**Results:**

After the LASSO and stepwise Cox regression analysis, the age, year of diagnosis, sites of SPMs, SEER stage of SPMs, surgery for SPMs, and radiotherapy for SPMs were included for model establishment. The ROC curve showed good discrimination for the 3- and 5-year AUC values in the training (0.774 and 0.779, respectively) and validation (0.758 and 0.763, respectively) cohorts. The calibration curve indicated good prognostic accuracy, especially in the 5-year survival prediction for this model. The DCA also demonstrated clinical efficacy over a wide range of threshold probabilities. Lastly, the risk group classified by the individual nomogram values showed significantly different survival outcomes in both training and validation cohorts.

**Conclusions:**

We constructed a nomogram to predict the OS of SPMs after HPC with good clinical values.

## 1. Introduction

Hypopharyngeal cancer (HPC) is a rare tumor that accounts for only 3%–5% of head and neck cancers [1, 2]. Due to the unique anatomical location, it is difficult to diagnose at an early stage, which leads to a poor prognosis [3], as evidenced by studies showing that 60% of HPC patients were first diagnosed at stage IV, and only 20% of HPC patients were first diagnosed at an early stage [4]. Furthermore, based on the fact that numerous HPC patients have had longstanding alcohol and tobacco consumption [5, 6], epithelial cells located on the aerodigestive tract, exposed to the carcinogen stimulations, are susceptible to genetic alterations followed by the onset of second primary malignancies (SPMs) [7, 8]. Moreover, according to field cancerization, the occurrence of head and neck cancers, including HPC, indicates the latent cancerization of the adjacent region, which increases the probability of SPMs [9]. With the popularization of endoscopic examinations, including tracheoscopy and gastroscopy, several studies reported the prevalence of SPMs in patients with head and neck cancer [10, 11]. It has been reported that about 15%–30% HPC patients has suffered from the SPMs [12]. Therefore, in addition to factors that are associated with the survival outcomes of HPC, such as local recurrence and distant metastasis of HPC, SPMs are also fundamental to assess the clinical outcome of HPC patients [13, 14]. Given the high heterogeneity of SPMs resulting from different sites, stages, histological classifications, and even therapy modalities, it is important to note that patients with SPMs have different prognoses. And follow-up visits included evaluation of symptoms, physical examination, endoscopy, computed tomography (CT), or magnetic resonance imaging (MRI) scans, which should be carried out every 3 months in the first 2 years, every 6 months between years 3 and 5, and once a year thereafter [15, 16].

Nomograms act as an effective method to predict the occurrence of clinical events and have been applied in survival prediction for multiple tumors [17–20]. However, there is still the absence of a systemic overview that focuses on HPC patients with SPMs due to the low prevalence of HPC. Therefore, in this study, we aimed to construct an overall survival nomogram model for this cohort of patients based on the Surveillance, Epidemiology, and End Results (SEER) database and corresponding advanced statistical methods. Findings from this study will help clinicians to evaluate the survival outcomes of HPC patients with SPMs according to their general clinical characteristics and select the optimal therapy modality.

## 2. Materials and Methods

### 2.1. Database and Patient Selection

Data were retrieved from the SEER Research Plus database using treatment modality information. A total of 1,174 patients diagnosed with HPC as the first primary cancer were extracted from the multiple primary-standardized incidence ratio (MP-SIR) sessions of SEER∗Stat version 8.3.8 (http://seer.cancer.gov/seerstat/).

Our exclusion criteria were as follows: (1) <18 years of age at diagnosis; (2) patients who had unknown information about disease characteristics, including histologic grade, SEER stage, and treatment modality of HPC and SPMs; and (3) a survival time of SPMs was 0 months. A time interval of at least 2 months was required between HPC and SPM diagnosis [21]. Finally, variables including demographic characteristics (year of diagnosis, age at diagnosis, sex, race, and marital status), disease characteristics (histologic grade, SEER stage, and site of SPMs), treatment modality (surgery, radiotherapy, and chemotherapy), and clinical outcomes for SPMs, such as the overall survival (OS), were collected.

### 2.2. Statistical Analysis

R statistical software version 4.1.0 (Bell Laboratories, Murray Hill, NJ, USA, downloaded from https://www.r-project.org/) was used to analyze the data.

The OS in our study was calculated according to the period from the date of SPM diagnosis to the date of the last follow-up or death in the SEER database. And cancer-specific survival (CSS) was calculated from the date of SPM diagnosis to the date of the last follow-up or death of cancer in the SEER database. Random sampling for our whole cohort of patients was achieved with the “sample” function in R software, and the patients were divided into training and validation cohorts at a ratio of 7 : 3, respectively. The least absolute shrinkage and selection operation (LASSO) regression was conducted using the “glmnet” package for all the variables we selected. Then, stepwise Cox regression was performed to build up the models, wherein the variables included were optimized under the lowest Akaike information criterion (AIC) value. Simultaneously, this Cox prognostic model for the 3-year and 5-year survival prediction was visualized by a nomogram generated by the “nomogram” function in the “rms” package. Cox regression was used to estimate hazard ratio (HR) and 95% confidence intervals (CI). The discrimination validation for this model was conducted using the receiver operating characteristics (ROC) curve, which was assessed by the area under the curve (AUC), and was calculated for the 3-year and 5-year survival in the training and validation cohorts, respectively. The decision curve analysis (DCA) was also utilized to assess the clinical efficacy of this model in both the 3-year and 5-year survival prediction. To assess the accuracy of this model, calibration curves were used to evaluate the calibration of the model at diverse time points, with 1,000 bootstrap resamples in both the 3-year and 5-year survival prediction.

The calculated sum score of each patient based on the nomogram was obtained by the “nomogram formula” package. According to the nomogram rankings of each patient in the training cohort, we stratified the training cohort into low-, medium-, and high-risk groups based on the first and second quartile values as cutoff points. Kaplan–Meier curves and log-rank tests were used to compare the OS of the patients in the different groups.

## 3. Results

### 3.1. Baseline Characteristics of Patients

**Figure 1:**
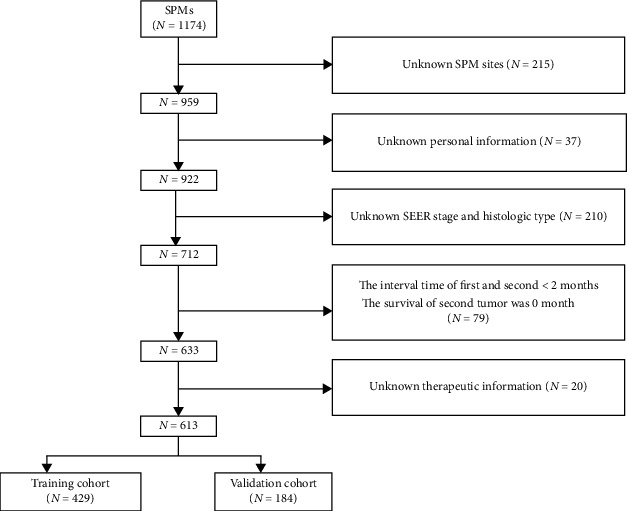
Flow diagram of selection for patients in our study. Abbreviation: SPMs: secondary primary malignancies.

The process of selection for the patients included in our study is shown in Figure 1. A total of 1,174 HPC patients with SPMs from 2000 to 2018 were extracted from the MP-SIR session in the SEER database. Among the 1,174 patients, the exclusion population included 215 patients (18.3%) without information on SPM sites, 37 patients (3.2%) without personal information such as marital status, 210 patients (17.9%) without SEER stage and histologic classification, 79 patients (6.7%) with less than 2-month intervals between HPC and SPMs or 0-month survival time, and 20 patients (1.7%) without therapeutic information. After exclusion, 613 HPC patients with SPMs were included in our study for model construction. Aided by the R software, the entire cohort was randomly divided into training and validation cohorts at a ratio of 7 : 3, respectively.

As shown in Table 1, there were no significantly different variables in the training and validation cohorts. In all cohorts, approximately 59.2% of patients were under 65 years of age, and 81.6% of the patients were male. In addition, 57.9% of the patients were married, and majority of the patients were white, accounting for about 81.6% of the patients. Regarding the time interval between HPC and SPMs, more than 24 months comprised majority of the patients (65.1%), and 26.9% of the patients were diagnosed between 2010 and 2018. Pyriform sinus (58.6%) was the major site of HPC. The majority of the HPC patients, with a proportion of approximately 98.4%, had squamous carcinoma (SCC), 65.3% of the patients had a regional site for the HPC compared to a localized and distant site in the SEER stage, and about 89.4% and 67.7% of the patients received radiotherapy and chemotherapy for HPC, respectively. However, only about 28.9% of patients received surgery, and 77.7% received at least two therapy models for HPC.

In terms of SPMs, the tumor sites were classified with the respiratory system, which had a proportion of 37.2%, digestive system, oral cavity/hypopharynx, and others. Approximately 48.8% of patients suffered from squamous SPMs, and the most frequent SEER stage of SPMs was localized, with an incidence of approximately 42.7%. Regarding the therapeutic aspect, 48.8% of patients received surgery for SPMs, 31.6% of patients received radiotherapy, and 29% received chemotherapy for SPMs. Regarding therapy modality, approximately 25.1% of patients experienced combined therapy. The site distribution of SPMs is shown in Table S1.

### 3.2. Prognostic Prediction Model Construction for the OS of SPMs

**Figure 2:**
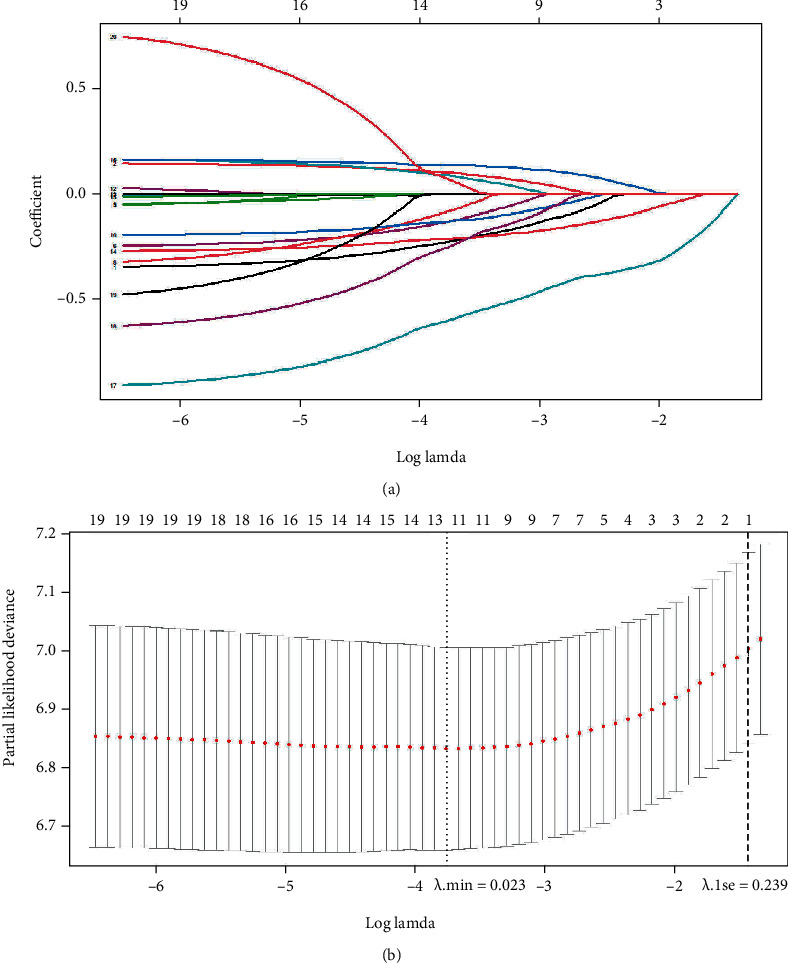
LASSO regression for exclusion of overfitting. (a) LASSO coefficient profiles of 20 variables for OS. (b) LASSO Cox analysis identified 11 variables for OS. Abbreviations: LASSO: least absolute shrinkage and selection operation; OS: overall survival.

We included 23 variables in the analysis, as shown in Figure 2(a). There were two cutoff values of *λ*, as shown in Figure 2(b), from which we selected *λ*_min_ to determine the 11 variables for next analysis, including “month intervals,” “age,” “race,” “year of diagnose,” “histology of HPC,” “surgery for HPC,” “sites of SPMs,” “SEER stage of SPMs,” “surgery for SPMs,” “radiotherapy for SPMs,” and “therapy modality for SPMs” (see Figure 2(b)). According to the concordance test, the *κ* value of the matrix was 6.36, indicating low multicollinearity compared to the *κ* value of 20.46 for the matrix before the LASSO regression.

**Figure 3:**
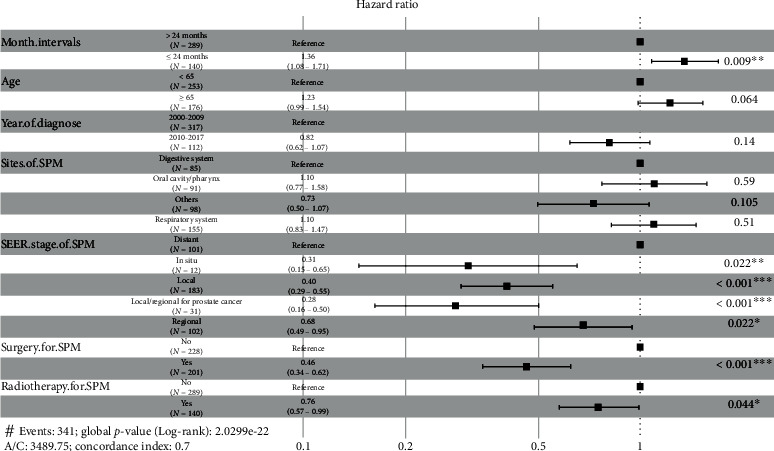
Forest plot for the Cox model. Abbreviation: SPMs: secondary primary malignancies.

**Figure 4:**
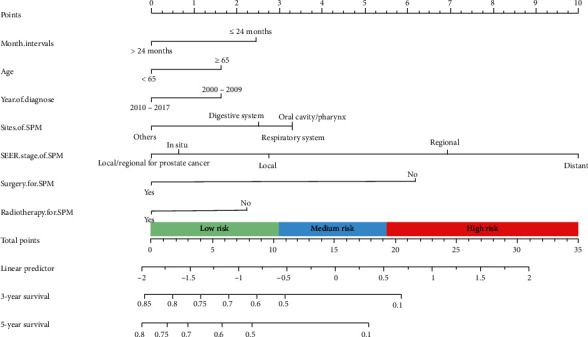
Nomogram for 3-year and 5-year OS from our model. Abbreviation: SPMs: secondary primary malignancies.

Then, stepwise Cox regression was conducted to further identify the variables for model construction; finally, seven variables identified with the lowest AIC value (3,489.75), which were “month intervals,” “age,” “year of diagnosis,” “sites of SPMs,” “SEER stage of SPMs,” “surgery for SPMs,” and “radiotherapy for SPMs,” were determined for model establishment. Through the LASSO regression and stepwise Cox regression for the exclusion of overfitting and multicollinearity, seven variables were included for the Cox model and nomogram, and the forest plot summary is shown in Figure 3; ≦24 months (HR: 1.36; 95% CI: 1.08–1.71, *P* = 0.009) was an independent risk factor. In situ (HR: 0.31; 95% CI: 0.15–0.65, *P* = 0.002), local (HR: 0.40; 95% CI: 0.29–0.55, *P* < 0.001), and local/regional for prostate cancer (HR: 0.28; 95% CI: 0.16–0.50, *P* < 0.001) and regional (HR: 0.68; 95% CI: 0.49–0.95, *P* = 0.022) relative to the distant SEER stage of SPMs were independent protective factors. In addition, surgery (HR: 0.46; 95% CI: 0.34–0.62, *P* < 0.001) and radiotherapy (HR: 0.76; 95% CI: 0.57–0.99, *P* = 0.044) for SPMs were also independent protective factors. The *C*-index of this Cox regression model was 0.704 (95% CI: 0.675–0.733). To visualize this model, a nomogram was constructed incorporating the seven variables to predict the 3-year and 5-year survival rates, as shown in Figure 4.

The LASSO regression of CSS is shown in Supplementary Figure 1, and the multivariate Cox analysis of CSS is shown in Table S2.

### 3.3. Validation of the Model

**Figure 5:**
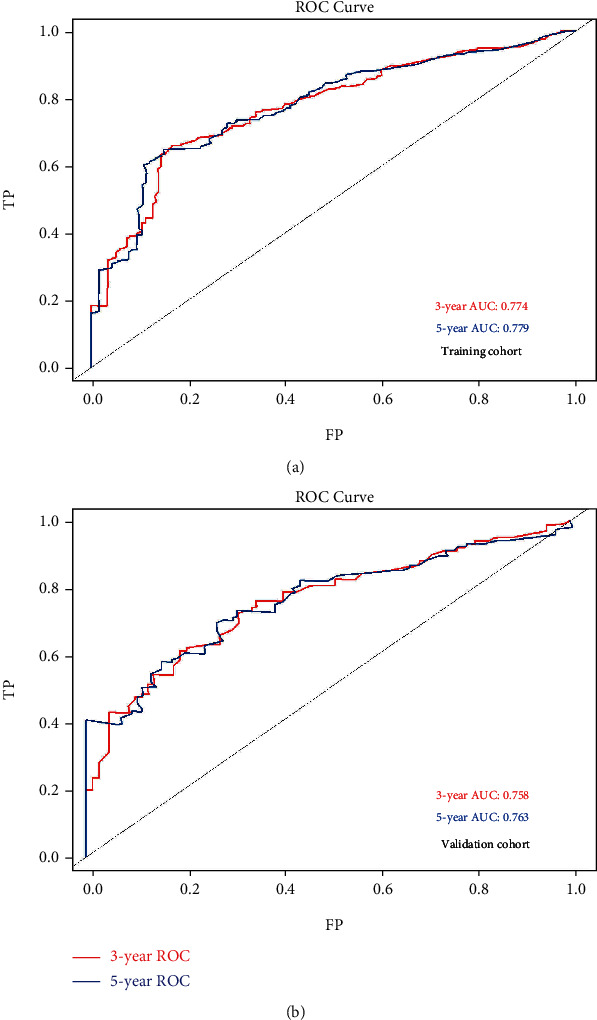
ROC curve for 3- and 5-year OS in (a) training cohort and (b) validation cohort with corresponding AUC values. Abbreviations: ROC: receiver operating curve; OS: overall survival.

**Figure 6:**
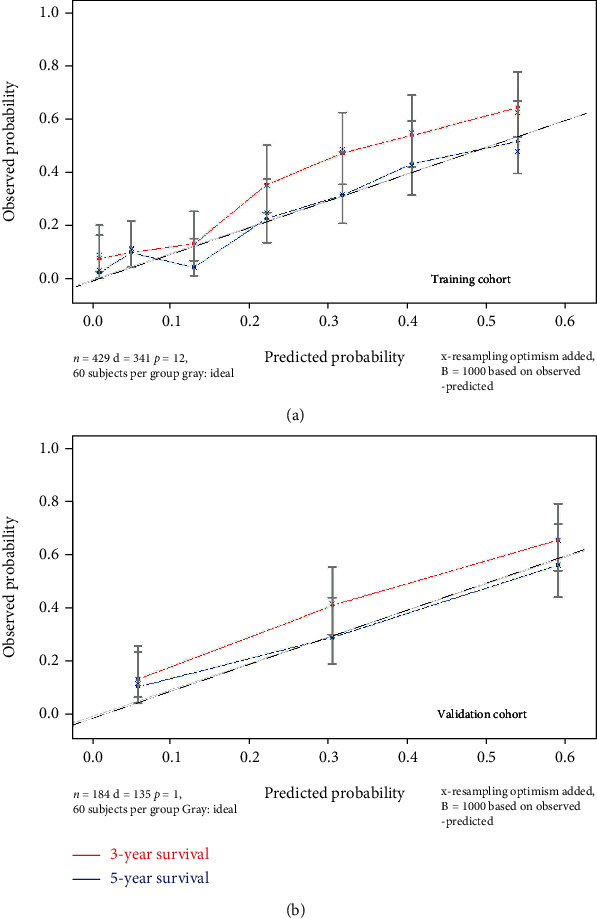
Calibration plots for predicting 3- and 5-year OS in (a) training cohort and (b) validation cohort. Abbreviation: OS: overall survival.

The validation process for this model was conducted for both training and validation cohorts. From the ROC analysis, the 3-year and 5-year AUCs were 0.774 and 0.779, respectively, in the training cohort (Figure 5(a)) and 0.758 and 0.763, respectively, in the validation cohort (Figure 5(b)), demonstrating the good discrimination of our model in the training and validation cohorts for both the 3-year and 5-year survival prediction. Moreover, the calibration curves displayed good consistency and accuracy, especially for the 5-year survival prediction (Figure 6(a)) in the training and validation cohorts (Figure 6(b)).

**Figure 7:**
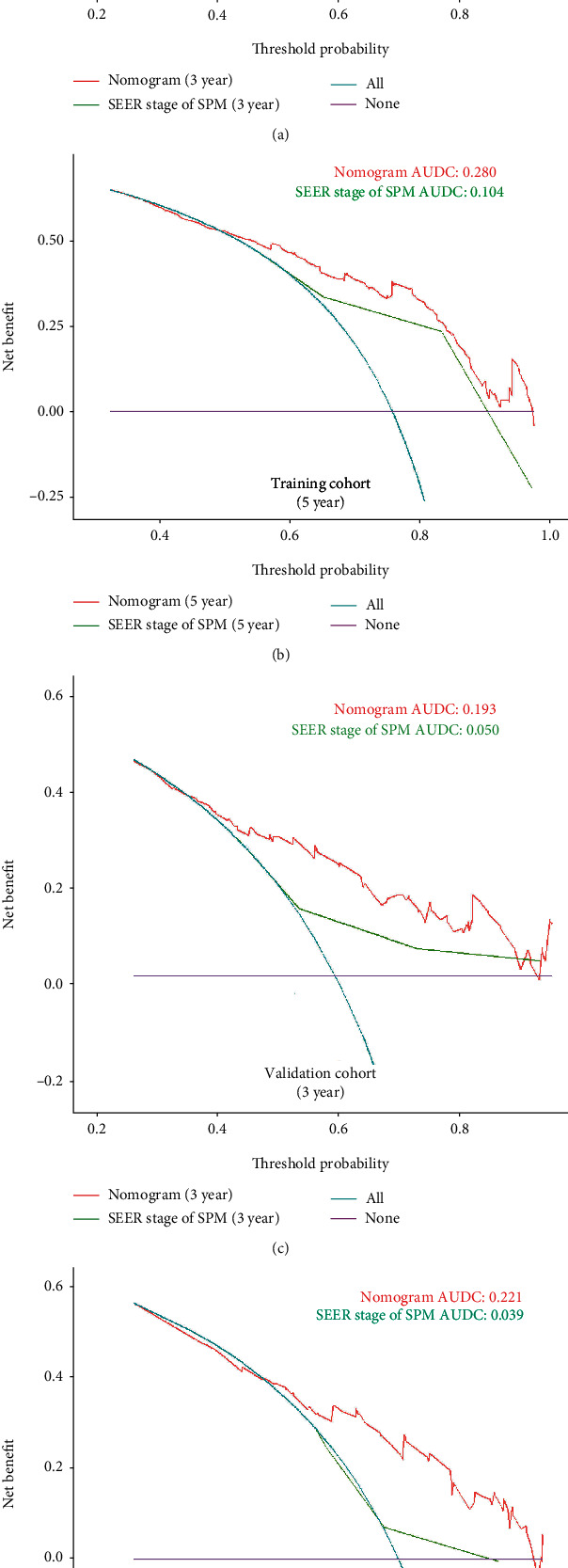
DCA curves of our nomogram and SEER stage of SPMs for (a) 3- and (b) 5-year OS in training cohort and (c) 3- and (d) 5-year OS in validation cohort. Abbreviations: DCA: decision curve analysis; SPMs: secondary primary malignancies; OS: overall survival.

Besides ROC analysis, DCA has been increasingly used for demonstrating the clinical efficacy of a clinical model. From the nomogram, the SEER stage of the SPMs was found to be a prominent factor for survival. Therefore, we constructed another control model using the SEER stage of the SPMs. According to the DCA curves of the 3-year and 5-year survival in the training and validation cohorts (Figure 7), our model (nomogram group) outperformed the SEER stage of the SPM-constructed model, with a wider range of threshold probability, leading to a positive net benefit and larger area under the decision curve (AUDC) in both the training (Figures 7(a) and 7(b)) and validation (Figures 7(c) and 7(d)) cohorts. In conclusion, all validation methods demonstrated good discrimination and accuracy of our model.

### 3.4. Risk Stratification of the HPC Patients with SPMs

**Figure 8:**
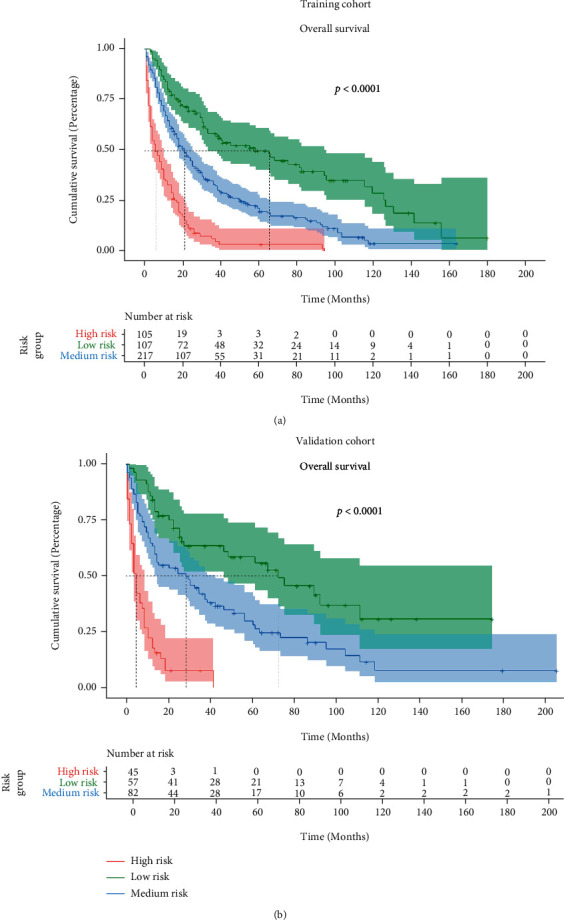
Kaplan–Meier curve for OS in (a) training cohort and (b) validation cohort for the patients stratified by the risk stratification system. Abbreviation: OS: overall survival.

Based on the nomogram for our model, the individual nomogram points were calculated in the training cohort, with values ranging from 2.759 to 27.455. We used the first quartile (10.780) and second quartile (19.470) to stratify the nomogram points into three groups: low-risk group, 2.759–10.780; medium-risk group, 10.780-19.470; and high-risk group, 19.470–27.455. The Kaplan–Meier survival analysis was then performed. In the training cohort, the median follow-up was 87.0 months (95% CI: 67.5–106.5 months), and the median, 3-year, and 5-year OS values were 65.0 months, 58.7%, and 50.1% in the low-risk group; 21.0 months, 33.6%, and 20.3% in the medium-risk group; and 6.0 months, 6.9%, and 4.2% in the high-risk group, respectively (*P* < 0.001, Figure 8(a)). In the validation cohort, the median follow-up was 82.0 months (95% CI: 0.7–103.3 months), and the median, 3-year, and 5-year OS values were 73.0 months, 63.5%, and 58.6% in the low-risk group; 29.0 months, 42.1%, and 29.9% in the medium-risk group; and 5.0 months, 7.9%, and 0% in the high-risk group, respectively (*P* < 0.001, Figure 8(b)), all of which implied the efficacy of our nomogram model to discriminate the different risk for the patients.

## 4. Discussion

In our study, the data extracted from the SEER database provided us with clinical information pertaining to both HPC and SPMs. A prognostic model for HPC with SPMs was established, and discrimination and calibration were assessed by AUC values in the ROC curve, calibration curve, and DCA curve as compared to those of the control model, suggesting the satisfactory performance of our model. Conversely, a risk stratification derived from our nomogram successfully distinguished the patients into different risk groups, supporting the feasibility and applicability of our model.

The 5-year AUC of the training cohort (0.779) and validation cohort (0.763) were similar with those of other studies [20], supposing the good discrimination of our study; in our study, the 5-year calibration outperformed the 3-year calibration, which could have been due to the relatively smaller size of the research cohort and longer survival period for the patients in our study.

Regarding the sites of SPMs for HPC, we found that the four sites of SPMs in the HPC patients were the lung and bronchus, oral cavity, prostate, and esophagus. This result corroborates previous reports suggesting that the lung and esophagus are the frequent sites of SPMs in head and neck cancer patients [22, 23]. Moreover, the lung was predisposed to be a metastasized site for head and neck cancer [24].

In our study, seven factors, including age, month intervals between the two tumors, histology of SPMs, SEER stage of SPMs, surgery, and radiotherapy for SPMs, were used for the model construction, in which longer month intervals predicted a better OS; the SEER stage of SPMs presented a strong negative correlation with the OS of SPMs, which was similar to those of other studies [25]. However, many researchers have shown that the stage system alone was not enough to predict the prognosis of patients [26, 27]; thus, we chose the SEER stage as the control model to confirm the efficacy of our model in the DCA curve.

The month interval was found to have a significant impact on prognosis, indicating that a month interval of less than 24 months worsened the survival. The reason for this phenomenon might be that the short time interval between the two cancers theoretically indicates severe field cancerization [28], eventually evolving into a highly aggressive SPMs.

In addition to age and stage, surgery and radiotherapy for SPMs were protective factors for survival; this result was consistent with many studies highlighting the importance of surgery and radiotherapy for the expected outcomes [29, 30]. However, chemotherapy was excluded, given that it does not have a protective role for OS; this may due to the acute and chronic toxicities caused by chemotherapy, counteracting the positive therapeutic effects. Another reason for this phenomenon might be that the patients without chemotherapy undergo surgery or chemotherapy. This indicates that, in contrast to patients with distant metastasis, proactive localized therapies exemplified with surgery and radiotherapy were necessary for SPMs and could be more advantageous than chemotherapy. Moreover, another report regarding the clinical prognosis of head and neck cancers demonstrated that patients without chemotherapy had a better prognosis [20].

Regarding the instructive significance of our model in clinical use, risk classification according to our nomogram could separate the whole cohort into different prognostic groups: favorable, intermediate, and poor prognosis groups. The patients classified into the low-risk group according to our model had an ideal prognosis, and enhanced therapy could potentially render these patients in a complete response. However, for patients classified into the medium- and high-risk groups using our model, clinicians should pay more attention. A trade-off between the toxicities of corresponding therapy and individual inclination should be considered for a more reasonable individualized therapy modality; as a result, prolonged survival and decent quality of life could be achieved.

This study was considered as a retrospective study, which is one of its limitations. Moreover, the differentiation status of tumors is a factor related to the prognosis of HPC [31]; however, owing to the lack of information on the differentiation of SPMs in HPC on the SEER database, the significance of the differentiation status in prognosis was not analyzed in our study. Moreover, the SEER database did not release the detailed chemotherapy modality and dose of radiotherapy; thus, we were not able to incorporate more factors related to therapy. Finally, considering that the patients included in our study were from 2000 to 2018, during which the American Joint Commission on Cancer (AJCC) stage had been updated from the 6^th^, 7^th^, to 8^th^ versions and that there was no unified standard to merge the different stage versions, the number of patients would be insufficient if only one version was adopted. Therefore, we had to substitute the AJCC stage with the SEER stage, which had similar effects on evaluating the stages of cancer.

## 5. Conclusions

In this study, we constructed the model and nomogram to predict the OS of HPC patients with SPMs and verified it using ROC, calibration plot, and DCA. Findings from our study show that this model could be used to predict the prognosis in 3-year and 5-year survival in a satisfactory discrimination, calibration, and clinical efficacy, while the risk stratification system according to the nomogram displayed an excellent indication capacity for the different risk groups. The variables determined to have effects on prognosis also conferred us with guidelines for better therapy and clinical management of HPC with SPMs.

## Figures and Tables

**Table 1:** Characteristics of hypopharyngeal cancer patients with SPMs.

Variable	Total	Training cohort	Validation cohort	*P*
	(*N* = 613)	(*N* = 429)	(*N* = 184)	
Age (y)				
<65	363 (59.2%)	253 (59.0%)	110 (59.8%)	0.858
≥65	250 (40.8%)	176 (41.0%)	74 (40.2%)	
Gender				
Female	113 (18.4%)	76 (17.7%)	37 (20.1%)	0.496
Male	500 (81.6%)	353 (82.3%)	147 (79.9%)	
Marital status				
Married	355 (57.9%)	247 (57.6%)	108 (58.7%)	0.858
Others	258 (42.1%)	182 (42.4%)	76 (41.3%)	
Race				
White	500 (81.6%)	349 (81.4%)	151 (82.1%)	0.983
Black	83 (13.5%)	59 (13.7%)	24 (13.0%)	
Others	30 (4.9%)	21 (4.9%)	9 (4.9%)	
Month interval (month)				
≤24	214 (34.9%)	140 (32.6%)	74 (40.2%)	0.079
>24	399 (65.1%)	289 (67.4%)	110 (59.8%)	
Diagnose year (y)				
2000-2009	448 (73.1%)	317 (73.9%)	131 (71.2%)	0.489
2010-2018	165 (26.9%)	112 (26.1%)	53 (28.8%)	
Primary site of HPC				
Pyriform sinus	359 (58.6%)	259 (60.4%)	100 (54.3%)	0.653
Postcricoid region	14 (2.3%)	10 (2.3%)	4 (2.2%)	
Aryepiglottic fold, hypopharyngeal	63 (10.3%)	43 (10.0%)	20 (10.9%)	
Posterior wall of the hypopharynx	42 (6.9%)	30 (7.0%)	12 (6.5%)	
Overlapping lesion of the hypopharynx	14 (2.3%)	10 (2.3%)	4 (2.2%)	
Hypopharynx, NOS	121 (19.7%)	77 (17.9%)	44 (23.9%)	
Histology of HPC				
SCC	603 (98.4%)	423 (98.6%)	180 (97.8%)	0.497
Others	10 (1.6%)	6 (1.4%)	4 (2.2%)	
SEER stage of HPC				
Localized	105 (17.1%)	76 (17.7%)	29 (15.8%)	0.775
Regional	400 (65.3%)	276 (64.3%)	124 (67.4%)	
Distant	108 (17.6%)	77 (17.9%)	31 (16.8%)	
Surgery for HPC				
No	436 (71.1%)	311 (72.5%)	125 (67.9%)	0.285
Yes	177 (28.9%)	118 (27.5%)	59 (32.1%)	
Radiotherapy for HPC				
No	65 (10.6%)	43 (10.0%)	22 (12.0%)	0.477
Yes	548 (89.4%)	386 (90.0%)	162 (88.0%)	
Chemotherapy for HPC				
No/unknown	198 (32.3%)	139 (32.4%)	59 (32.1%)	1
Yes	415 (67.7%)	290 (67.6%)	125 (67.9%)	
Therapy model for HPC				
None or alone	137 (22.3%)	98 (22.8%)	39 (21.2%)	0.674
Combined therapy	476 (77.7%)	331 (77.2%)	145 (78.8%)	
Tumor site of SPM				
Respiratory system	228 (37.2%)	155 (36.1%)	73 (39.7%)	0.716
Digestive system	122 (19.9%)	85 (19.8%)	37 (20.1%)	
Oral cavity and pharynx	123 (20.1%)	91 (21.2%)	32 (17.4%)	
Others	140 (22.8%)	98 (22.8%)	42 (22.8%)	
Histology of SPM				
SCC	299 (48.8%)	210 (49.0%)	89 (48.4%)	0.973
Adenocarcinoma	178 (29.0%)	125 (29.1%)	53 (28.8%)	
Others	136 (22.2%)	94 (21.9%)	42 (22.8%)	
SEER stage of SPM				
In situ	16 (2.6%)	12 (2.8%)	4 (2.2%)	0.725
Localized	262 (42.7%)	183 (42.7%)	79 (42.9%)	
Regional	154 (25.1%)	102 (23.8%)	52 (28.3%)	
Distant	137 (22.3%)	101 (23.5%)	36 (19.6%)	
Localized/regional (only for prostate)	44 (7.2%)	31 (7.2%)	13 (7.1%)	
Surgery for SPM				
No	314 (51.2%)	228 (53.1%)	86 (46.7%)	0.159
Yes	299 (48.8%)	201 (46.9%)	98 (53.3%)	
Radiotherapy for SPM				
No	419 (68.4%)	289 (67.4%)	130 (70.7%)	0.449
Yes	194 (31.6%)	140 (32.6%)	54 (29.3%)	
Chemotherapy for SPM				
No	435 (71.0%)	305 (71.1%)	130 (70.7%)	0.923
Yes	178 (29.0%)	124 (28.9%)	54 (29.3%)	
Therapy model for SPM				
None or alone	459 (74.9%)	323 (75.3%)	136 (73.9%)	0.761
Combined	154 (25.1%)	106 (24.7%)	48 (26.1%)	

Abbreviations: SPMs: second primary malignancies; HPC: hypopharyngeal cancer; SCC: squamous cell carcinoma.

## Data Availability

Data are available upon reasonable request from the corresponding authors.

## Supplementary Materials

Supplementary 1 Supplementary Figure 1: LASSO regression for cancer-specific survival. Abbreviations: LASSO: least absolute shrinkage and selection operation.

Supplementary 2 Table S1: the site distribution of SPMs.

Supplementary 3 Table S2: multivariate Cox regression for cancer-specific survival.
