# Supplementary figures and images for: A Novel Overall Survival Nomogram Prediction of Secondary Primary Malignancies after Hypopharyngeal Cancer: A Population-Based Study
Source: J Oncol. 2022 Apr 28;2022:4681794. doi: 10.1155/2022/4681794 (PMC9073552; doi:10.1155/2022/4681794)

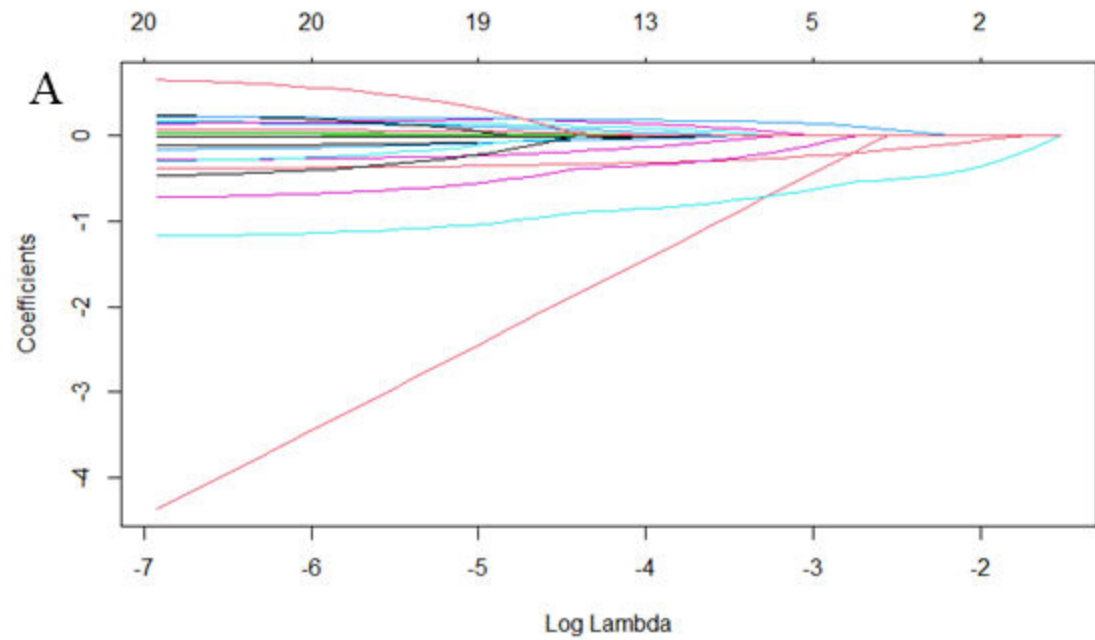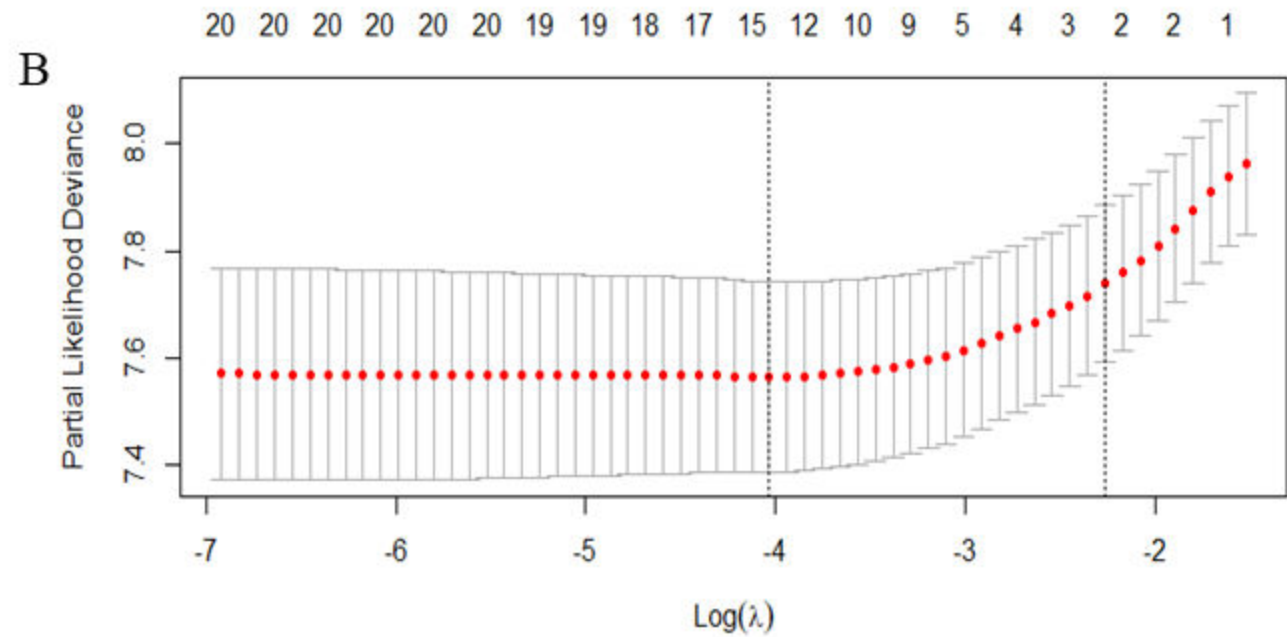

Supplement: Supplementary 1 — Supplementary Figure 1: LASSO regression for cancer-specific survival. Abbreviations: LASSO: least absolute shrinkage and selection operation. [file 4681794.f1.pdf]
