# Supplementary material for: A Novel Overall Survival Nomogram Prediction of Secondary Primary Malignancies after Hypopharyngeal Cancer: A Population-Based Study
Source: J Oncol. 2022 Apr 28;2022:4681794. doi: 10.1155/2022/4681794 (PMC9073552; doi:10.1155/2022/4681794)
Supplement: Supplementary 2 — Table S1: the site distribution of SPMs. [file 4681794.f2.docx]

| **Table S1: The sites distribution of SPMs** | |
| --- | --- |
| **Sites of SPMs** | **Number** |
| Lung and Bronchus | 226 |
| Oral cavity | 67 |
| Prostate | 59 |
| Esophagus | 45 |
| Pharynx | 34 |
| Colon and rectum | 29 |
| Larynx | 25 |
| Urinary Bladder | 22 |
| Liver | 16 |
| Kidney | 15 |
| Stomach | 8 |
| Breast | 7 |
| Pancreas | 7 |
| Melanoma of the Skin | 7 |
| Thyroid | 6 |
| Leukemia | 6 |
| Myeloma | 4 |
| Cecum | 4 |
| Other Non-Epithelial Skin | 4 |
| Corpus Uteri | 2 |
| Intrahepatic Bile Duct | 2 |
| Vulva | 2 |
| Gallbladder | 2 |
| Other Oral Cavity and Pharynx | 2 |
| Other Digestive Organs | 1 |
| Peritoneum, Omentum and Mesentery | 1 |
| Other Biliary | 1 |
| Ovary | 1 |
| Nose, Nasal Cavity and Middle Ear | 1 |
| Other Male Genital Organs | 1 |
| Nasopharynx | 1 |
| Renal Pelvis | 1 |
| Soft Tissue including Heart | 1 |
| Cranial Nerves Other Nervous System | 1 |
| Splenic Flexure | 1 |
| Penis | 1 |
| Abbreviations: SPMs: second primary malignancies | |
