# Supplementary material for: A Novel Overall Survival Nomogram Prediction of Secondary Primary Malignancies after Hypopharyngeal Cancer: A Population-Based Study
Source: J Oncol. 2022 Apr 28;2022:4681794. doi: 10.1155/2022/4681794 (PMC9073552; doi:10.1155/2022/4681794)
Supplement: Supplementary 3 — Table S2: multivariate Cox regression for cancer-specific survival. [file 4681794.f3.docx]

| **Table S2. Multivariate Cox regression for cancer-specific survival** | | |
| --- | --- | --- |
| Variable | HR (95% CI) | *P* |
| **site of SPM** |  |  |
| Respiratory system | Reference |  |
| Digestive system | 0.729 (0.529-1.005) | 0.054 |
| Oral cavity and pharynx | 1.155 (0.828-1.610) | 0.397 |
| Others | 0.419 (0.271-0.649) | <0.001 |
| **SEER stage of SPM** |  |  |
| In situ | Reference |  |
| Localized | 1.238 (0.500-3.066) | 0.644 |
| Regional | 1.871 (0.750-4.667) | 0.179 |
| Distant | 4.138 (1.655-10.347) | 0.002 |
| Localized/regional (only for prostate) | 0.993 (0.328-3.010) | 0.990 |
| **Surgery for SPM** |  |  |
| No | reference |  |
| Yes | 0.437(0.326-0.585) | <0.001 |
| Abbreviations: SPMs: second primary malignancies. | |  |
